# The Impact of Homelessness on Lung Cancer Survival and Healthcare Utilization in the Hungarian Universal Healthcare System

**DOI:** 10.3390/cancers17071158

**Published:** 2025-03-29

**Authors:** Daniel Heilig, Ákos Szabó, Petra Fadgyas-Freyler, Judit Simon

**Affiliations:** 1 Department of Health Economics, Center for Public Health, Medical University of Vienna, 1090 Vienna, Austria; daniel.heilig@meduniwien.ac.at; 2 Department of Financial Accounting, Corvinus University of Budapest, 1093 Budapest, Hungary; 3 Department of Health Policy, Corvinus University of Budapest, 1093 Budapest, Hungary

**Keywords:** homelessness, lung cancer, survival analysis, healthcare costs, public health

## Abstract

Differences in the lifestyle and health-service-seeking behavior of persons experiencing homelessness (PEH) are well known. However, a comprehensive estimation of the resulting healthcare utilization differences and health outcome impacts are so far under-researched, especially at the national level. We aim to close this gap of evidence for lung cancer within the Hungarian universal healthcare system. We analyzed lung cancer-related information for the PEH population in the Hungarian national central health insurance register dataset and a matched control population between 2015 and 2021. In this period, 11,857 people were registered as homeless for the majority of at least one year. To capture the effect of homelessness, we created a categorical “homelessness length index” (HLI) according to the proportion of time an individual experienced homelessness during the investigation period (HLI 0: 0; HLI 1: >0–1/3; HLI 2: >1/3–2/3; HLI 3: >2/3). PEH individuals were matched 1:5 with a non-PEH control sample adjusted for age, sex and region. We conducted Kaplan–Meier survival analysis and Cox proportional hazards (CPH) regression adjusted for age, sex, HLI and average lung cancer-related healthcare costs. Our final analysis sample consisted of 641 patients (233 PEH, 408 control) who were newly diagnosed with lung cancer during the observation period. The lung cancer prevalence in the PEH group was 1.97% as opposed to 0.69% in the control group. The Kaplan–Meier curve showed lower average survival times for the PEH group, compared to the control group. Lung cancer associated costs between diagnosis and death/censoring were substantially different with average per patient cumulative costs of EUR 3668 in the PEH group compared to EUR 6827 in the control group (2018 prices). This translated to an average 47% lower annual lung cancer-related healthcare cost per PEH patient. CPH analysis showed that after disease severity, the degree of time spent in homelessness had the most significant effect on mortality, with a HR of 1.47 associated with both HLI 2 and 3 (95% CI: 1.08–2.00 and 1.01–2.14, respectively). We find that in Hungary, homelessness is associated with a much higher lung cancer burden linked to a three-times-higher prevalence, lower overall survival and almost 50% lower annualized disease-specific healthcare costs. Worse outcomes are mainly driven by long-term homelessness. Results could not be further refined according to lifestyle-related factors due to data availability limitations.

## Simple Summary

This study looks at the health effects of homelessness in subjects diagnosed with lung cancer in a nationwide dataset in Hungary. Healthcare costs and survival analysis showed worse outcomes, with the length of experienced homelessness playing a key role.

## 1. Introduction

Many studies have noted the detrimental health effects of homelessness. A recent review and meta-analysis covering related studies found worse outcomes for people experiencing homelessness (PEH) in virtually all disease fields and all-cause mortality [1]. The definitions for homelessness can include different types but most broadly, a PEH is defined as a person lacking a fixed, adequate nighttime residence [2]. Nationwide studies on the health effects of homelessness, however, are virtually nonexistent due to homelessness being an issue that is rarely found in national databases with widespread coverage, due to PEH having no official insurance status or claim. This generally does not mean PEH receive no health care, but rather that examining healthcare utilization is not feasible. This study is based on Hungarian health insurance data with the unique opportunity to quantify the effects of homelessness on disease outcomes and healthcare utilization due to universal health coverage at the national level including PEH.

Lung cancer is the most common cancer-related cause of death worldwide, according to the World Health Organization (WHO) [3]. It has similar high mortality rates for both sexes but shows differences in outcomes across the socioeconomic spectrum [4]. It is also the most common cancer in the homeless population of a previous study [5]. These factors make it a suitable disease to compare survival outcomes between PEH and a control population. If a difference in survival is observed, it is also important to investigate the likely causes of this difference as far as possible in the given data.

Hungary has a single-payer universal healthcare system, where the National Health Insurance Fund (NHIF) provides high (around 95%) population coverage to roughly 9.7 million inhabitants [6]. This Bismarckian social insurance system requires an entitlement reason (and preferably a contribution payment) for insurance coverage such as employment, retirement status, student status, etc. One of these entitlement reasons can be homelessness. In order to gain this homeless status, providers specializing in care for PEH have the right to register their service users in the NHIF database, thus ensuring them full insurance coverage for the next six months [7]. All insured individuals have a unique patient identifier received at birth or by entering the national health insurance system at a later time point (e.g., foreign citizens employed in Hungary). This identifier does not change during an individual’s lifetime and is linked to their place of residence and, if applicable, the death date from the central registries. This system provides the possibility to track all registered PEH in Hungary at a national level over an extended period of time and observe the time spent in homelessness, as well as all health services received in the Hungarian health insurance system.

To see what impact homelessness has on lung cancer care costs and overall survival outcomes in a universal healthcare system such as Hungary, we conducted a retrospective cohort study with administrative healthcare data provided by the Hungarian NHIF.

## 2. Materials and Methods

### 2.1. Data Source

The central NHIF dataset contains records for all publicly reimbursed health services used by insured individuals, including primary care, specialist outpatient care, inpatient care, accident and emergency care, transport, pharmaceuticals, medical imaging, etc. Due to the type of reimbursement, fee-for-service (FFS) in the form of a so-called German point system mixed with Diagnosis-Related Groups (DRGs) for acute inpatient care, the NHIF database contains ICD-10 (International Statistical Classification of Diseases and Related Health Problems 10. Revision) diagnostic codes, admission-related DRGs and ATC (Anatomical Therapeutic Chemical Classification) pharmaceutical codes at an individual patient-level. Through linkage with the death registry, the recorded date of death of each individual who died in the observed period is also available. Due to death registry data being mostly available without cause-of-death information, all survival analyses in this study reflect all-cause mortality.

### 2.2. Study Sample

**Figure 1:**
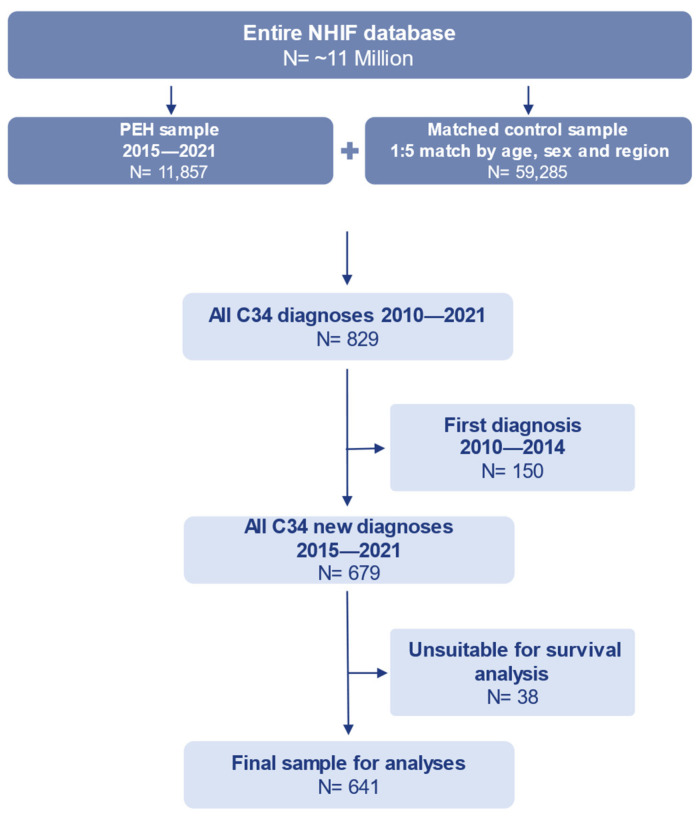
Study sample.

For our study sample, we selected all individuals from the NHIF database, whose typical entitlement (>6 months) reason was homelessness in any given year between 2015 and 2021 (PEH sample). We then created a matched control group of the same sex and age from the same region of residence with a one-to-five case-to-control ratio (control sample). Our study thus draws lung cancer cases from the resulting total (Figure 1). We then compared these two samples for newly diagnosed lung cancer outcomes and costs. Consequently, data from all persons out of the homeless and matched populations who had a diagnosis of lung cancer between 2010 and 2021 were extracted for analysis. Diagnoses of lung cancer were defined as any healthcare services received for a main diagnosis of C34 according to the ICD-10 classification system [8]. Cases of lung cancer between 2010 and 2014 were only used to filter for newly diagnosed cases from 2015 onwards, without a previous record of a C34 diagnosis. They were not used in the main analysis since we wanted to capture the entire disease progression as far as possible including survival times and received healthcare services from first diagnosis to death alongside the time spent in homelessness. Furthermore, some lung cancer cases newly diagnosed after 2015 had to be excluded because their lung cancer diagnoses were dated after their registered death dates (Figure 1). This is most likely due to the cancer having been found during autopsy, since Hungary has one of the highest autopsy rates in Europe [9].

We stratified the sample according to the share of time spent in homeless status between 2015 and 2021. For this, a categorical variable called the homelessness length index (HLI), was created where Category 0 stands for no time spent as homeless, i.e., the control cohort. Categories 1, 2 and 3 of the HLI were defined as up to 1/3, >1/3 up to 2/3 and >2/3 of the total observation time spent as homeless, respectively. The decision for thirds was made after sensitivity testing different groupings of the share of time. Overall, stratification by thirds showed the most homogenous groups with the most stable differences between groups. This stratification also proved to have the best model fit. To judge the model fit in comparison, we used the concordance index as proposed by Harrel et al. [10,11]. This final three-stage categorization also aligns with the three-group typology for homelessness by Kuhn and Culhane [12] representing transitional, episodic and chronic homelessness.

For health service utilization costs, all costs that were registered in the database linked with the diagnosis code for lung cancer (C34) were summed up on an individual patient level annually in 2018 prices. The discounting of later years was performed according to the rates given by the UNECE Statistical Database [12]. All cost data used in this analysis were given in Hungarian Forint (HUF), so there was no need to approximate them via assigned DRG points as would be the norm in many other healthcare insurance datasets. Furthermore, to compare these costs to other healthcare systems, we converted them into Euros and purchase power parity-adjusted Euros (PPPEUR) by using the appropriate exchange rates and PPP conversion factors given by the UNECE and Eurostat [13,14]. In the NHIF dataset, costs are split into five categories: inpatient, pharmaceuticals, outpatient, radio-diagnostics and “other”. “Other costs” contains item-based medicine and item-based devices, implants and procedures. These assets are expensive and, therefore, in the case of some products (e.g., medicaments without Health Technology Assessment (HTA) approval) require special approval (compassionate use). Some chemotherapy products are also included in this category.

### 2.3. Statistical Analyses

For survival outcomes, we compared median survival times and five-year survival and graphically plotted survival curves using the Kaplan–Meier approach [15]. Survival times were calculated from the date of the index lung cancer diagnosis until the recorded date of death, if available, or the date of censoring otherwise. All individuals still at risk at the end of 2021 were right censored. Furthermore, we restricted the maximum observation period to 5 years for all individuals since that was the longest period with sufficient data in both groups for stratification.

To take into account the effects of other parameters on survival such as age, sex and healthcare cost differences, we calculated the Cox proportional hazards (CPH) regression [16]. This multivariate regression approach is the most widely used method to infer the effect of different variables on survival outcomes in medical research [17]. The average yearly healthcare cost was also used as a control variable to explore the extent of mortality hazard differences that could be associated with differential healthcare resource use between the PEH and control groups. We conducted the CPH analyses in a stepwise approach by adding more variables one by one into different models and rerunning the analysis. Better-performing variables that described the same characteristics were chosen. Since no staging information was available, we used metastatic cancer codes as a proxy for more severe stages of lung cancer. To identify these, we flagged all patients where C77–C79 codes were registered in addition to the initial lung cancer diagnosis. The confidence interval was set to 95% and a *p* value of <0.05 was considered statistically significant. All analyses were conducted using R statistical software version 4.1.1. This study was based on the official data provision process of the National Health Insurance Fund of Hungary [18] (approval number: I043-46-2023, date of approval: 5 April 2023). Since data were handled directly on the NHIF servers and only aggregated secondary data were published here, the study was exempt from additional ethics approval.

## 3. Results

### 3.1. Sample Descriptives

For the period 2015–2021, there were 11,857 individuals who were classified as having homeless status (PEH sample) and 59,285 individuals in the matched control cohort. In the PEH sample, 23% (n = 2762) were females. Lung cancer was the most common cancer observed, with a prevalence of approximately 20% (Appendix A, Table A1).

Of the total 71,142 individuals in the analysis sample, 641 were newly diagnosed with lung cancer during the observation period (Figure 1). There were 233 PEH cases (PEH group) compared to 408 control cases (control group) corresponding to prevalence rates of 1.97% and 0.69%, respectively (Table 1).

Considering homelessness, lung cancer cases were overrepresented in the higher HLI groups with a prevalence of 3.06% in the HLI 3 group, 2.31% in the HLI 2 group and 1.58% in the HLI 1 group (Table 1).

### 3.2. Healthcare Costs

We summed up all healthcare costs associated with a C34 diagnosis between the index diagnosis and death or censoring. This amounted to an average of HUF 1,136,568 per lung cancer case in the PEH group and HUF 2,115,777 per lung cancer case in the control group, translating into a 46% lower average total lung cancer treatment cost per PEH patient. These costs corresponded to EUR 3564 and EUR 6635 (2018 prices) and to PPP EUR 12,052 and PPP EUR 22,436 (2018 prices), respectively [13]. The difference in annualized cost per patient was similarly 47% less in the PEH group (Table 2). The inpatient costs were the most similar: only 20% lower per patient in the PEH group. In contrast, pharmaceutical costs were the most different, over 80% lower per PEH patient, although these costs were only a minor fraction of the total costs for both groups (3% for PEH vs. 8% for control). A considerable part of lung cancer costs was recorded in the “other costs” category (49% for PEH vs. 59% for control) (Table 2).

### 3.3. Survival

**Figure 2:**
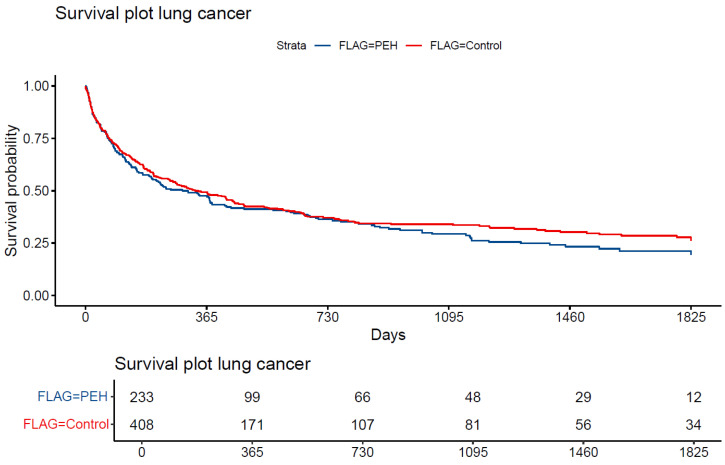
Kaplan–Meier survival plot (*p* = 0.3).

**Figure A1:**
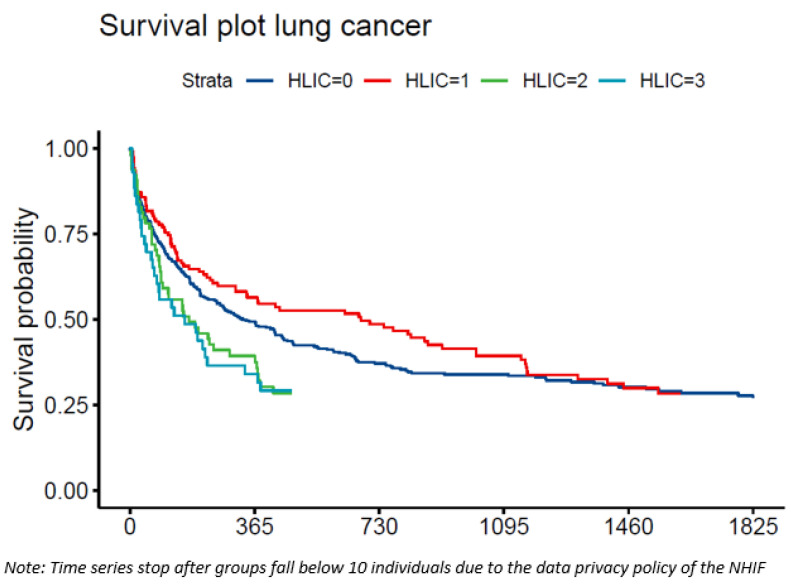
KM survival plot by HLI category (*p* = 0.004).

Figure 2 shows the Kaplan–Meier survival curves stratified by group. After an initial big drop in survival representing the high mortality rates of certain lung cancers in the first year, the Kaplan–Meier survival curves of the two groups converged before further clear separation after two years (Figure 2). The median survival times were 292 days (95% CI: 200–419) for the PEH group and 330 (95% CI: 419–447) for the control group. The difference when only looking at a single homelessness category, however, was not statistically significant (*p* = 0.3). Kaplan–Meier survival analysis by HLI category was only possible for the first year due to sample size issues. This showed a clear separation of HLI 2 and 3 from HLI 0 and 1, meaning that persons experiencing long-term, chronic homelessness showed a significantly lower initial average survival time when compared to no or episodic homelessness (Appendix A, Figure A1). To test this observed difference and measure its magnitude, we used CPH regression analysis.

All-cause mortality hazard ratios (HRs) were tested for significance for different CPH models, including different homelessness stratification approaches and control variables. We found significant interactions between all-cause mortality and age, HLI, lung cancer-specific healthcare costs and metastatic cancer stages. As shown by the rising concordance index, the model performance improved with each variable addition after settling on the most fitting way to stratify the sample by the level of homelessness.

First, we controlled for age and sex in all six models shown in the table. Although female sex showed a reduced HR, this did not reach statistical significance in our study. Nevertheless, based on previous evidence about sex-based differences in lung cancer survival and the potential magnitude of female sex impact in our data, sex remained included as a standard control variable in all models [20,21]. Age showed a significant association with all-cause mortality (HR = 1.04, 95% CI 1.02–1.05) that was robust across all models. Being one year older increased the hazard of dying in the given period by 4%, keeping all other factors constant (Models 4–6) (Table 3).

Models 2 and 3, which included homelessness as a dummy or continuous variable, did not deliver significant results as opposed to Model 4 where homelessness experience was stratified based on the aforementioned sensitivity analysis and literature [12]. While compared to the control cohort, short-term homelessness (HLI 1: 59% of the PHE group) showed no significant difference, mortality hazard increased significantly by more than 50% for episodic or long-term homelessness represented in our sample by HLI 2 and 3 (HR = 1.59, 95% CI: 1.18–2.16; and HR = 1.66, 95% CI: 1.15–2.41, respectively). Between HLI 2 and 3, the HRs only differed slightly when controlled for other factors (Models 5 and 6) (Table 3).

To control for costs in the model, we included annualized lung cancer-associated healthcare costs on an individual level. Higher costs were associated with a significantly lower mortality hazard (−0.03 per additional HUF 100,000). However, the overall magnitude of this association was only marginal when compared to the associations with longer-term homelessness or disease severity (Models 5 and 6).

Metastatic cancer had by far the strongest association with mortality differences. With an HR of 2.37 (95% CI: 1.92–2.93), patients with metastatic cancer diagnoses were more than twice as likely to die at any point during the observation period than those without. Furthermore, the differential results between Models 4 to 6 indicate that in total about 25–40% of the excess mortality hazard for HLI 2 and 3 was explainable by lower lung cancer-specific healthcare costs and a higher prevalence of metastatic cancer cases in these groups (Table 3).

## 4. Discussion

Generally, the Hungarian population of PEH we covered in this study is similar to those in other studies; for example, the percentage of females in our study’s PEH population (23%) is in line with the average sex distribution in homeless populations according to a recent systematic review [22].

Our study shows that homelessness is not only associated with an increase in lung cancer prevalence for the Hungarian PEH population, but this increase is linked to the magnitude of time spent in homelessness. The high general prevalence and it being the most common cancer in the PEH population is in line with a previous study [5].

This higher prevalence is likely to be due to multiple factors. First, there is usually a much higher smoking rate amongst PEH populations [23]. Consequently, a previous study found excess rates of many smoking attributable cancer types in the homeless population of Boston [24]. A second factor that has been proposed to influence lung cancer prevalence is pulmonary tuberculosis infections, being up to five times as prevalent among PEH as in the non-homeless population [25,26]. Since our study was based on administrative data, we could not control for any of these factors.

Our cost estimates of EUR 3668 and EUR 6827 for PEH and controls (EUR 5519 between both groups) are along the lines of the EUR 4157 average treatment costs for lung cancer in Hungary from Inotai et al. based on earlier data from 2000 to 2012 [27]. Our average treatment cost results of PPP EUR 18,661 also compare to the respective treatment costs of EUR 25,063, EUR 17,777 and EUR 32,500 from France, England and Germany, as described by McGuire et al. in 2015 [28].

We found only a limited positive impact of higher healthcare costs on lowering mortality hazard, which may be reflective of the high fatality rate and standardized treatment patterns for lung cancer. These results may also simply reflect the increased treatment costs associated with a longer survival time. Overall, we found only limited evidence that survival differences between PEH and controls with lung cancer are caused by significantly different healthcare utilization. Our differential survival results are rather indicative of more metastatic cancer cases within the PEH group, most likely due to later diagnosis and lower general ability to battle lung cancer, especially in the case of episodic and long-term homelessness (HLI 2 and 3). 

A previous study in the USA found that a deficit in quality of life, as experienced by PEH, lowered overall survival [29] and that from a survival perspective, the main focus should lie on preventing people from experiencing long-term homelessness [30]. Although we were not able to reflect on quality of life, our findings are in line with this conclusion, showing that transitional or short-term homelessness (HLI 1) had no significant impact on survival, while longer-term homelessness increased the mortality hazard by around 50%. These findings also support the hypothesis that a large proportion of socio-economic differences in survival, which have been observed in other studies summarized by Redondo-Sánchez et al. [4], are the result of differences in lifestyle and general resilience rather than experienced differences in treatment.

It has to be noted though that these findings may only be generalizable to universal healthcare systems similar to those in Hungary where PEH can have full access to public healthcare services. In less equitable healthcare systems, it is likely that a similar analysis, if feasible, would yield different results. From a public health perspective, however, Hungary’s policy of providing PEH with full access to public healthcare services seems to influence survival to some degree with the median survival time of 292 days among PEH with lung cancer when compared to a study from Seattle, where the PEH lung cancer patient median survival time was 211 days [31].

Further research may expand this study by looking at different cancer types and other chronic diseases in the context of homelessness and outcomes. It would also be important to assess whether previously experienced homelessness retains its legacy effect on disease outcome and survival even if a certain time spent out of homelessness has passed.

Our study is based on administrative health insurance data, which brings some general data limitations with it. Firstly, no data on privately funded healthcare service use are available in the NHIF database. For the current study, it is very unlikely that homeless people would have used private services and paid out-of-pocket, but this may not be true for the control group, which can lead to some downward bias in the magnitude of health service utilization differences. On the other hand, since the majority of lung cancer treatment is conducted in inpatient or outpatient specialist care settings, relevant services would be covered in the dataset as standard. A second general limitation is due to known up-coding activities as a consequence of FFS reimbursement [32,33]. However, these biases would appear in both groups (PEH and control) equally and, therefore, would not affect the comparative results of the analyses. The third important administrative limitation of our dataset is the potential selection bias of homeless cases. Since PEH status is registered when accessing some type of health or care service provision, not all homeless individuals in Hungary are registered by the NHIF. Those who appear in the NHIF dataset are likely to be in a more fragile health status requiring acute care. Yet again, in the case of lung cancer, this bias may not be of particular importance in comparison to some chronic diseases. Lastly, due to the ICD code format, we were not able to reflect on cancer staging information and the potentially differential impacts of small- and non-small-cell lung cancers on survival, healthcare utilization and cost differences that may also drive some of the group differences [34]. However, we tried to catch some of these differences by controlling for metastatic cancer codes which coincided with lung cancer diagnoses.

## 5. Conclusions

The results of this study imply that equitable healthcare provision for diagnosed lung cancer cases may only influence negative survival outcome differences faced by homeless people to a certain degree. The evidence on prevalence, metastatic stages and survival differences indicates that the lung cancer disease burden is considerably higher in the PEH group, which on average should lead to higher yearly costs with no difference in access. The lower average costs for the PEH group, as seen in this case study for lung cancer from Hungary, however, show that mere access does not necessarily lead to higher utilization. Additional emphasis on screening, early detection and tackling issues that lead to long-term homelessness is also needed to improve outcomes for this vulnerable group. Earlier detection and treatment could not only improve survival and quality of life but also limit the downstream costs of more severe lung cancer cases. Overall, our findings support the need for broader, more complex policies to improve healthcare utilization among PEH with lung cancer.

## Figures and Tables

**Table 1:** Sample characteristics.

	All		New Lung Cancer Cases	
	PEH Sample	Control Sample	PEH Group	Control Group
Total (N/%)	11,857 (17%)	59,285 (83%)	233 (36%)	408 (64%)
Males (N/%)	9095 (77%)	45,475 (77%)	196 (82%)	372 (85%)
Females (N/%)	2762 (23%)	13,810 (23%)	43 (18%)	68 (15%)
Age in 2015 (mean ± SD)	43 (±12)	43 (±12)	53 (±7)	55 (±7)
Males	44 (±12)	44 (±12)	53 (±7)	56 (±7)
Females	41 (±12)	41 (±12)	53 (±6)	53 (±6)
Age at C34 diagnosis (mean ± SD)	-	-	56 (±7)	58 (±7)
Males	-	-	56 (±7)	58 (±7)
Females	-	-	56 (±6)	56 (±6)
Number of years registered as homeless (mean ± SD)	2.1 (±1.5)	-	1.9 (±1.3)	-
Percentage of time registered as homeless (mean ± SD)	33% (±25)	-	40% (±29)	-
Homelessness Length Index (HLI, N/%):				
HLI 1	8095 (68% ^†^)	-	138 (59% ^‡^)	-
HLI 2	2292 (19% ^†^)	-	52 (22% ^‡^)	-
HLI 3	1470 (12% ^†^)	-	43 (19% ^‡^)	-

^†^ % refers to the entire PEH sample (N = 11,857). Percentages are rounded to the nearest full number and do not add up to 100%. ^‡^ % refers to a PEH sample with a lung cancer diagnosis (N = 233).

**Table 2:** Lung cancer associated healthcare costs (in 2018 prices, HUF and PPP EUR).

	HUF		EUR		PPP EUR		Difference PEH vs. Control	Percentage of Total Costs	
	PEH	Control	PEH	Control	PEH	Control		PEH	Control
Average C34 associated **total** healthcare costs per patient	1,136,568	2,115,777	3564	6635	12,052	22,436	−46%	100%	100%
Average C34 associated **total** healthcare costs **per patient year**	675,562	1,276,978	2119	4004	7163	13,541	−47%	100%	100%
Average C34 associated **inpatient** costs **per patient year**	295,223	366,925	926	1151	3131	3890	−20%	44%	29%
Average C34 associated **pharmaceuticals** costs **per patient year**	19,119	100,084	60	314	203	1,061	−81%	3%	8%
Average C34 associated **outpatient** costs **per patient year**	15,008	26,245	47	82	159	278	−43%	2%	2%
Average C34 associated **radio-diagnostics** costs **per patient year**	15,410	29,328	48	92	163	311	−47%	2%	2%
Average C34 associated **other** costs **per patient year**	330,802	754,397	1037	2366	3508	8000	−56%	49%	59%

Source: NHIF data, exchange rates from Eurostat [14] PPP EUR rates from OECD [19].

**Table 3:** Cox proportional hazards (CPH) regression results.

CPH	HR (95% CI)					
	Model 1	Model 2	Model 3	Model 4	Model 5	Model 6
Age at diagnosis	1.03 (1.01–1.05) *	1.03 (1.01–1.05) *	1.03 (1.01–1.05) *	1.03 (1.02–1.05) *	1.04 (1.02–1.05) *	1.03 (1.02–1.05) *
Female sex	0.83 (0.63–1.02)	0.83 (0.63–1.02)	0.83 (0.63–1.01)	0.88 (0.67–1.16)	0.89 (0.67–1.18)	0.92 (0.69–1.21)
Homelessness yes/no	-	1.02 (0.99–1.47)	-	-	-	-
Homelessness in years	-	-	0.99 (0.91–1.07)	-	-	-
HLI						
1	-	-	-	0.96 (0.75–1.24)	0.98 (0.76–1.26)	1.03 (0.80–1.32)
2	-	-	-	1.59 (1.18–2.16) *	1.51 (1.11–2.05) *	1.47 (1.08–2.00) *
3	-	-	-	1.66 (1.15–2.41) *	1.50 (1.03–2.18) *	1.47 (1.01–2.14) *
Total annualized lung cancer associated healthcare costs (per HUF 100,000)	-	-	-	-	0.97 (0.96–0.98) *	0.97 (0.96–0.98) *
Metastatic cancer	-	-	-	-	-	2.37 (1.92–2.93) *
Concordance index	0.563	0.565	0.563	0.584	0.675	0.690

HR: hazard ratio. Bold * = significant variables at *p* < 0.05.

## Data Availability

Due to the sensible nature of the NHIF individual health data, the underlying data can only be described in aggregate form and are not available to the general public. Requests to access the data should be directed to the NHIF.

## Appendix A

**Table A1:** Ten most common cancers by case percentage in the PEH population.

Cancer ICD 10 Code	Percentage of All Cancer Cases
C34	19.8%
C79	6.4%
C32	6.0%
C78	5.9%
C77	4.6%
C10	3.4%
C53	3.1%
C13	2.9%
C18	2.5%
C67	2.5%
